# Interim PET-CT may predict PFS and OS in T-ALL/LBL adult patients

**DOI:** 10.18632/oncotarget.19572

**Published:** 2017-07-26

**Authors:** Liang Wang, Jing-Hua Wang, Xi-Wen Bi, Xiao-Qin Chen, Yue Lu, Zhong-Jun Xia

**Affiliations:** ^1^ Department of Hematologic Oncology, Sun Yat-sen University Cancer Center, Guangzhou, 510060, China; ^2^ State Key Laboratory of Oncology in South China, Collaborative Innovation Center for Cancer Medicine, Guangzhou, 510060, China; ^3^ Department of Medical Oncology, Sun Yat-sen University Cancer Center, Guangzhou, 510060, China

**Keywords:** T lymphoblastic leukemia/lymphoma, PET-CT, international harmonization project criteria, prognosis

## Abstract

T lymphoblastic leukemia/lymphoma (T-ALL/LBL) is highly aggressive. Although intensive chemotherapies such as ALL-type regimens are commonly used, about half adult patients eventually relapse and die of T-ALL/LBL. Overwhelming evidences have confirmed that interim PET can predict survival outcomes and guide subsequent treatments in Hodgkin lymphoma. However, whether interim PET-CT can predict survival outcomes or not in T-ALL/LBL patients remains unclear. 47 adult patients of T-ALL/LBL were retrospectively reviewed. Interim PET-CT was done after induction therapy and evaluated according to the International Harmonization Project criteria. After induction therapy, interim PET-CT was positive in 19 patients (40.4%). After a median follow up time of 34 months, the 2-year and 3-year progression free survival (PFS) rate were 39% and 30%, respectively, and the 2-year and 3-year overall survival (OS) rate were 54% and 45%, respectively. Using Kaplan-Meier survival analysis, it was found that interim PET-CT positivity correlated with significantly inferior PFS and OS (2-year PFS rate for patients with positive or negative interim PET were 21.1% or 56.0%, respectively, *p* = 0.002; 2-year OS rate for patients with positive or negative interim PET were 31.6% or 63.7%, respectively, *p* = 0.010). However, there was no significant relationship between PFS, OS and bone marrow infiltration, lactate dehydrogenase level, and stages (*p* > 0.05). Interim PET-CT may predict PFS and OS in adult patients of T-ALL/LBL, which needs to be validated in prospective clinical trials. The optimal criteria for interim PET-CT evaluation and risk-adapted treatment strategy determined by interim PET-CT should be investigated in future clinical practice.

## INTRODUCTION

T-lymphoblastic lymphoma (T-LBL) is a relatively rare type of lymphoma, and accounts for about 1.7% of all adult patients with non-Hodgkin lymphoma (NHLs) [1]. T-LBL is considered to arise from thymic T cells, and there is a great overlap between T-LBL and T-cell acute lymphoblastic leukemia (T-ALL) concerning both the biological and clinical features [2], resulting in the general recognition that T-LBL and T-ALL are the same disease with different stages. Thus, T-LBL and T-ALL were combined together as “T-ALL/LBL” in the World Health Organization (WHO) classification in 2008. The commonly used chemotherapy regimens for NHLs were considered to be unsuitable for T-ALL/LBL due to relatively low complete remission rate and dismal long-term survival outcomes [3–7]. Whereas patients of T-ALL/LBL treated with ALL-type regimens achieved improved outcomes [8].

Combination of 18-F-fluorodeoxyglucose-positron emission tomography (PET) and computed tomography (CT), PET-CT, is a very sensitive and specific imaging technique for both patients with solid tumors and various types of lymphoma. Many studies have confirmed that interim PET can predict survival outcomes in Hodgkin lymphoma (HL) [9–11] and interim PET-guided treatment strategies have been applied in clinical trials of HL [11–14]. Recently, increasing evidence has proved that interim PET-CT can also predict survival outcomes in diffuse large B cell lymphoma (DLBCL) [15–19]. PET-CT is helpful in determining the nature of residual mass, especially distinguishing between viable tumor and necrosis or fibrosis. T-ALL/LBL is usually featured by mediastinal bulky mass [2], and cannot shrink to normal or disappear completely after treatment in some patients, for whom it is considered that PET-CT will be useful in assessing the treatment response in mediastinum. There are few literatures concerning interim PET-CT in predicting survival outcomes in patients of T-ALL/LBL. Thus, we retrospectively analyzed the results of interim PET-CT in adult patients of T-ALL/LBL treated with ALL-type intensive chemotherapies.

## RESULTS

### Patients

The patients’ characteristics are shown in Table 1. The male to female ratio was 3:1, and the median age at diagnosis was 26 years old (range 17–65), with 29.8% of patients being older than 30 years old. About 90% of patients had mediastinal mass at presentation and 53.2% had bone marrow infiltration, with the median percentage of bone marrow (BM) blast cells being 25% (1%–90%). 85.1% of patients had advanced disease.

**Table 1:** Patients’ characteristics

Parameters	*N* = 47 (%)
Gender	
Male	35 (74.5)
Female	12 (25.5)
Age	
> 30	15 (29.8)
≤ 30	32 (70.2)
ECOG PS score	
≥ 2	11 (23.4)
< 2	36 (76.6)
B symptoms	
Yes	19 (40.4)
No	28 (59.6)
Stage	
I–II	7 (14.9)
III–IV	40 (85.1)
Bone marrow infiltration	
Yes	25 (53.2)
No	22 (46.8)
LDH level	
Elevated	16 (34.0)
Normal	31 (66.0)
Mediastinal mass	
Yes	42 (89.4)
No	5 (10.6)
Chemotherapy regimens	
ECOG 2993	5 (10.6)
BFM-90	27 (57.4)
HyperCVAD/MA	12 (25.5)
unspecified	3 (6.5)
Interim PET-CT	
Positive	19 (40.4)
Negative	28 (59.6)

Abbreviations: ECOG, Eastern Cooperative Oncology Group; PS, performance status; LDH, lactate dehydrogenase; HyperCVAD/MA, fractionated cyclophosphamide, vincristine, doxorubicin, and dexamethasone, alternating with high-dose methotrexate and cytarabine therapy.

### Interim PET-CT results and treatment responses

**Figure 1:**
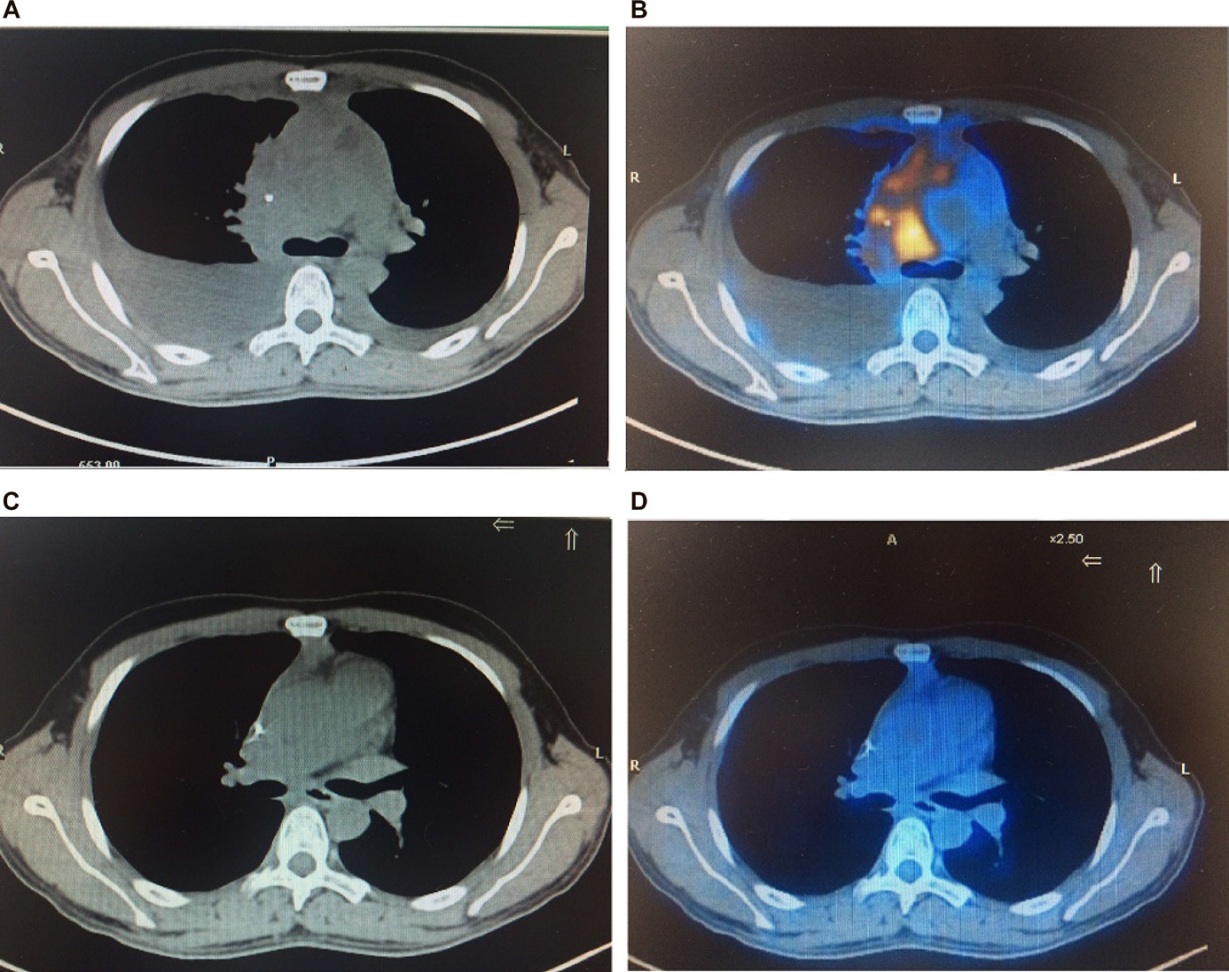
Baseline and interim PET-CT scans for a patient of T-LBL Baseline CT (**A**) and PET (**B**) scan revealed a mediastinal mass and moderate pleural effusion. Interim PET-CT scan (**C** and **D**) after induction therapy confirmed CR.

**Figure 2:**
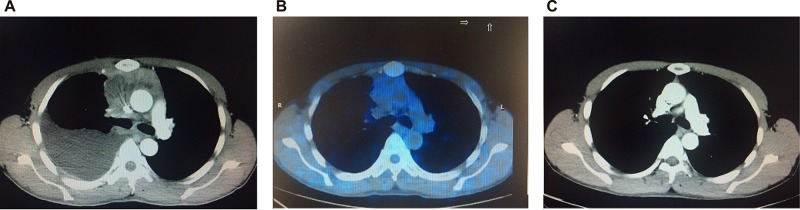
Imaging scans through the whole treatment for a patient of T-LBL Baseline CT scan (**A**) revealed a mediastinal mass and moderate pleural effusion. Interim PET-CT scan (**B**) showed positivity with residual lesion in the mediastinum. CT scan (**C**) after consolidation therapy confirmed CR.

After induction therapy (i.e. after the end of phase 1a or 1b or 2 cycles of hyperCVAD/MA), interim PET-CT was positive in 19 patients (40.4%), most of whom had residual disease in the mediastinal lesion, and negative in 28 patients (59.6%) (Figure 1). Among those 19 patients with PET-CT positivity, 12 patients had BM infiltration at diagnosis and all of them got BM CR after induction therapy, thus subsequent consolidation therapy was given according to their respective regimens. 2 of those 19 patients received salvaged nelarabine treatment, with no response in both patients. Subsequent consolidation therapy was given according to their respective regimens and CR was achieved in one patient (Figure 2). The remaining 5 patients did not receive any further treatment in our center. After consolidation therapy or 6 cycles of hyperCVAD/MA, another 10 patients got PET-CT negativity or unconfirmed CR by CT scan, resulting in a CR rate of 80.9% (including the 5 patients who abandoned treatment) or 90.5% (excluding the 5 patients who abandoned treatment). Among the 28 patients who had interim PET-CT negativity, bone marrow CR was achieved in all patients. However, 9 patients relapsed at a median of 10 months (range, 3–20 months), with bone marrow relapse in 6 patients and mediastinal lesion relapse in 3 patients.

### Survival analysis

**Figure 3:**
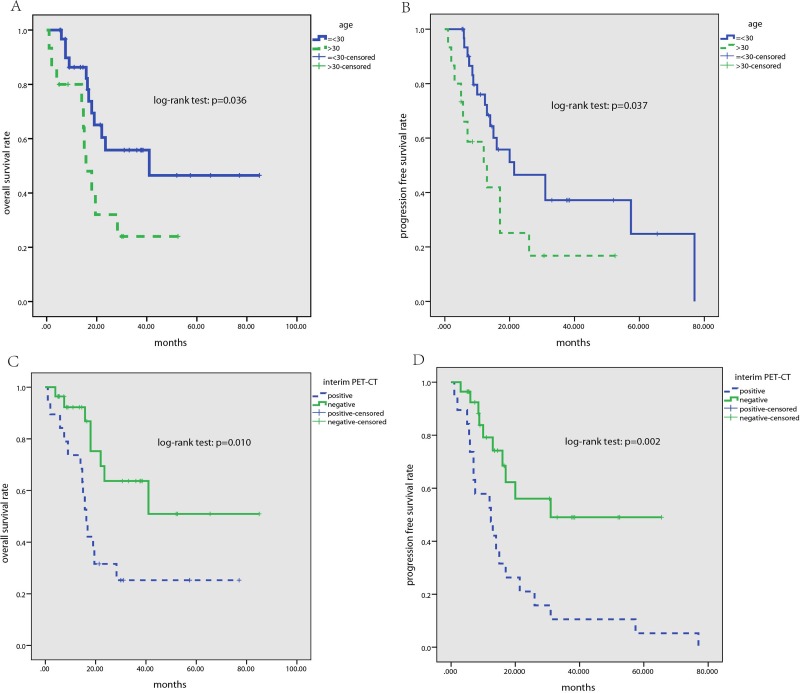
Kaplan-Meier survival analysis for all patients with T-ALL/LBL (**A**) 3-year OS rate for patients with age ≤ 30 and > 30 was 55.8% and 24.0%, respectively (*p* = 0.036); (**B**) 3-year PFS rate for patients with age ≤ 30 and > 30 was 37.2% and 16.8%, respectively (*p* = 0.037); (**C**) 3-year OS rate for patients with interim PET-CT negativity and positivity was 63.7% and 25.3%, respectively (*p* = 0.010); (**D**) 3-year PFS rate for patients with interim PET-CT negativity and positivity was 49.0% and 10.5%, respectively (*p* = 0.002).

After a median follow up time of 34 months, 29 patients had disease progression or relapse, of which 22 patients died of the disease, resulting in the 2-year and 3-year progression free survival (PFS) rate being 39% and 30%, respectively, and the 2-year and 3-year overall survival (OS) rate being 54% and 45%, respectively. Using Kaplan-Meier survival analysis and log-rank test, it was found that patients with higher age (> 30) had inferior PFS and OS than younger patients (*p* = 0.037 and 0.036, respectively). Furthermore, interim PET-CT positivity correlated with significantly inferior PFS and OS (2-year and 3-year PFS rate for patients with positive or negative interim PET-CT were 21.1% and 10.5%, or 56.0% and 49.0%, respectively, *p* = 0.002; 2-year and 3-year OS rate for patients with positive or negative interim PET-CT were 31.6% and 25.3%, or 63.7% and 63.7%, respectively, *p* = 0.010) (Figure 3). However, there were no significant relationship between PFS, OS and BM infiltration, lactate dehydrogenase level, stages, and treatment regimens (*p* > 0.05).

## DISCUSSION

Lymphoblastic lymphoma is a highly aggressive neoplasm of lymphoblasts of either B- (B-LBL) or T-cell origin (T-LBL), combined together with ALL in the 2008 WHO classification of hematopoietic malignancies. These two entities are biologically very close but not identical, especially in terms of clinical presentations. T-LBL is usually featured by a large mediastinal mass with no or minimal BM involvement, while in T-ALL, all patients have BM involvement and typically present with a high lymphocyte count. However, the differentiation between T-ALL and T-LBL is arbitrary, with a figure of > 25% BM blasts being used as the threshold for defining T-ALL. Due to inferior survival outcomes, lymphoma-like regimens have been abandoned in the treatment of T-ALL/LBL [3–5]. Modern LBL protocols are based on intensive multi-drug ALL-like regimens, including an intensive remission induction chemotherapy, an early central nervous system(CNS) prophylaxis, consolidation blocks, and subsequent maintenance therapy [20].

Treatment decision should be made tailored to the individual patient, so the early identification of factors that can predict treatment outcomes in T-ALL/LBL is important to improve survival outcomes. International Prognostic Index (IPI) has been widely used as a predictor of prognosis in lymphoma patients, however, it might not accurately reflect treatment outcomes because of disease heterogeneity between individuals [21]. Therefore, there is an urgent need to develop new tools to predict treatment outcomes early during the treatment.

Until now, most of the tumor responses induced by chemotherapy are assessed by morphological imaging modalities, such as CT. However, changes of metabolic function often precede changes of anatomical structures; thus, it is sometimes difficult to determine the efficacy by analyzing anatomical images only, especially when tumor lesions are replaced by fibrosis and/or tumor necrosis without a considerable shrinkage in volume [22, 23]. PET with the radiotracer ^18^F-FDG is widely used for staging and restaging of DLBCL and HL [24], but may also be used to provide an early prediction of the effectiveness of therapy [10, 16, 18]. Currently, evaluation of the interim response using PET-CT is considered predictive of early relapse in HL [10]. Studies of interim PET-CT in DLCBL, however, have yielded controversial results [15, 18, 25]. We conducted this retrospective study to define whether interim PET-CT can be used for survival prediction or not in patients of T-ALL/LBL.

Our study focused on a cohort of patients of T-ALL/LBL treated with ALL-like regimens, and an interim PET-CT scan was performed after induction phase or 2 cycles of hyperCVAD/MA. We found that interim PET-CT had prognostic value in terms of PFS and OS. In addition, we found that patients with higher age (> 30) had inferior PFS and OS than younger patients, which was consistent with previous study [7]. After a median follow up time of 34 months, the 2-year and 3-year PFS rate were 39% and 30%, respectively, and the 2-year and 3-year OS rate were 54% and 45%, respectively. The survival of the patients in our study seems poorer than previously reported [7, 26–28]. More patients with advanced stage and higher incidence rate of bone marrow invasion in our study may contribute to this finding. Moreover, none of the patients in our study received auto-HSCT or allo-HSCT. In this study, 28 patients had interim PET-CT negativity, of which 9 patients relapsed at a median of 10 months (range, 3–20 months), with bone marrow relapse in 6 patients and mediastinal lesion relapse in 3 patients. The limitation of PET sensitivity in bone marrow minimal residual disease and false negativity of interim PET may explain why patients with negative interim PET-CT still relapse.

There are some limitations in this study. Firstly, we did not perform biopsies from interim PET-CT positive residual masses due to unconvenience or potential risks (most residual masses located in mediastinum), and false positivity may exist. Secondly, our treatment regimens were not uniform. Finally, subsequent treatment regimens were not changed according to the interim PET-CT results. Thus, our results need to be validated in prospective clinical trials. However, the significant inferior outcomes in patients with positive interim PET-CT suggest changing subsequent treatment regimens for those patients may be considered in further clinical trials.

In conclusion, this study confirms that interim PET-CT may predict PFS and OS in patients of T-ALL/LBL treated with ALL-type regimens. As such, interim PET-CT may be used to tailor subsequent therapy with the aim of maximizing cure rate while minimizing unnecessary toxicity. In an era of promising alternative chemotherapies, emerging antibodies, and biotherapies, patients with positive interim PET-CT may benefit from an early change in therapeutic approach. However, larger prospective trials and optimization and standardization of criteria for interim PET-CT evaluation are needed to assess the real prognostic value of interim PET-CT in T-ALL/LBL. Furthermore, more correlation studies between the interim PET-CT and other known biomarkers should be conducted.

## MATERIALS AND METHODS

### Patients

115 consecutive patients diagnosed as T-ALL/LBL were treated in the department of Hematologic Oncology of Sun Yat-sen University Cancer Center from January 2000 to July 2014, of whom interim PET-CT results and detailed follow-up information were available in 47 patients. All these patients were diagnosed according to the WHO Classification of Tumours of Haematopoietic and Lymphoid Tissues. Patients with significant bone marrow (BM) infiltration were also included in this study.

### Treatments and response evaluation

All patients received ALL-type treatment regimens. 27 patients were treated with modified Berlin-Frankfurt-Münster (BFM)-90 regimen as previously reported [28]. 12 patients received hyperCVAD/MA (fractionated cyclophosphamide, vincristine, doxorubicin, and dexamethasone, alternating with high-dose methotrexate (HD-MTX) and cytarabine therapy) regimen as previously reported [26]. Medical Research Council (MRC) UKALLXII/Eastern Cooperative Oncology Group (ECOG) 2993 regimen [29] was given in 5 patients and unspecified intensive regimens were administrated in 3 patients. All these patients did not receive cranial prophylactic radiotherapy, but at least eight times of regular intrathecal chemotherapy were given. For patients treated with modified BFM-90 regimen, four additional high dose methotraxate treatments were given during maintenance therapy. None of those 47 patients received autologous stem cell transplantation (auto-HSCT) or allogeneic stem cell transplantation (allo-HSCT). Clinical response was evaluated at the end of each phase of the protocol (For patients received BFM-90 and 2993 regimens, response evaluation was done after the end of phase 1a of induction therapy and phase 1b of induction therapy, at the end of the consolidation course and at the completion of therapy; for patients treated with hyperCVAD/MA, response evaluation was done after each cycle of treatment). Patients with BM complete remission (CR) and no significant residual disease after induction therapy were given consolidation therapy according the respective regimens. Patients who did not get BM CR or with significant residual disease were given salvage therapy such as nelarabine. Follow-up visit was scheduled every 3 months for the first 2 years after treatment and then every 6 months during the next 5 years. The definition of CR, PR, and relapse was as previously reported [28].

### Interim PET-CT

All 47 patients underwent interim PET-CT scan after induction therapy phase (phase 1a or phase 1b) or 2 cycles of hyperCVAD/MA. Patients fasted for at least 6 hours before the PET-CT scan, and blood-sugar level was checked before the exam. ^18^F-FDG was administered intravenously. A whole-body acquisition was started 60 minutes after a 2 to 5 MBq/kg F-18 FDG injection on a dedicated CT-PET camera. All images were interpreted visually by an experienced nuclear physician according to the International Harmonization Project (IHP) criteria. A negative PET-CT scan was defined as having no residual abnormal uptake or a minimal residual uptake. A positive scan was defined as having at least one residual site associated with intensity markedly superior to the local background.

### Statistical analyses

Overall survival (OS) was calculated from the date of diagnosis until the date of death as a result of any cause, censoring at the time of the last follow-up. PFS was calculated from the date of diagnosis until disease relapse, censoring at the time of last follow-up. Survival probabilities were calculated by using the Kaplan-Meier method and compared using log-rank tests. Differences were considered statistically significant at a *P* value of less than 0.05. Analyses were performed using SPSS version 19 statistical software.

### Ethics

All procedures performed in this study were in accordance with the ethical standards of the institutional and/or national research committee and with the 1964 Helsinki Declaration and its later amendments or comparable ethical standards. For this retrospective study, formal consent is not required.
